# Transcriptome and Metabolome Analysis of Rice Cultivar CBB23 after Inoculation by *Xanthomonas oryzae* pv. *oryzae* Strains AH28 and PXO99^A^

**DOI:** 10.3390/plants13101411

**Published:** 2024-05-18

**Authors:** Pingli Chen, Junjie Wang, Qing Liu, Junjie Liu, Qiaoping Mo, Bingrui Sun, Xingxue Mao, Liqun Jiang, Jing Zhang, Shuwei Lv, Hang Yu, Weixiong Chen, Wei Liu, Chen Li

**Affiliations:** 1 Guangdong Key Laboratory of New Technology in Rice Breeding, Guangdong Rice Engineering Laboratory, Key Laboratory of Genetics and Breeding of High Quality Rice in Southern China (Co-Construction by Ministry and Province), Ministry of Agriculture and Rural Affairs, Rice Research Institute, Guangdong Academy of Agricultural Sciences, Guangzhou 510640, China; 2 Guangzhou Academy of Agricultural Sciences, Guangzhou 510335, China

**Keywords:** *Xanthomonas oryzae*, *Xa23*, AH28, RNA-seq, metabolome

## Abstract

Bacterial leaf blight (BLB), among the most serious diseases in rice production, is caused by *Xanthomonas oryzae* pv. *oryzae* (*Xoo*). *Xa23*, the broadest resistance gene against BLB in rice, is widely used in rice breeding. In this study, the rice variety CBB23 carrying the Xa23 resistance gene was inoculated with AH28 and PXO99^A^ to identify differentially expressed genes (DEGs) associated with the resistance. Transcriptome sequencing of the infected leaves showed 7997 DEGs between the two strains at different time points, most of which were up-regulated, including cloned rice anti-blight, peroxidase, pathology-related, protein kinase, glucosidase, and other coding genes, as well as genes related to lignin synthesis, salicylic acid, jasmonic acid, and secondary metabolites. Additionally, the DEGs included 40 cloned, five NBS-LRR, nine SWEET family, and seven phenylalanine aminolyase genes, and 431 transcription factors were differentially expressed, the majority of which belonged to the WRKY, NAC, AP2/ERF, bHLH, and MYB families. Metabolomics analysis showed that a large amount of alkaloid and terpenoid metabolite content decreased significantly after inoculation with AH28 compared with inoculation with PXO99^A^, while the content of amino acids and their derivatives significantly increased. This study is helpful in further discovering the pathogenic mechanism of AH28 and PXO99^A^ in CBB23 rice and provides a theoretical basis for cloning and molecular mechanism research related to BLB resistance in rice.

## 1. Introduction

Rice is among the most important food crops, providing food for more than half of the global population. Bacterial leaf blight (BLB), which is caused by the gram-negative bacterium *Xanthomonas oryzae* pv. *oryzae* (*Xoo*), is one of the most important diseases that harm rice production [1]. It has the characteristics of strong mutability and fast transmission and inoculation rates, and outbreaks are difficult to control effectively. Therefore, cultivating and planting new varieties with high and broad-spectrum resistance is the most economical and effective method for controlling BLB [2], and constantly exploring new genes for excellent resistance is the basis and key to the success of resistance breeding.

Transcription activator-like effectors (TALEs) are the most important vir factors of *Xoo*, and the type III secretion system (T3SS) injects TALE proteins into rice cells. Under the action of rice intracellular transporters, the TALEs ultimately enter the host nucleus and specifically bind to the effector-binding element (EBE) sequence of the target gene promoter region. Plant resistance to disease is manipulated through the transcriptional activation of target gene expression [3]. In the long-term rice–*Xoo* co-evolution process, in order to avoid manipulation by pathogens, the natural variation or artificial mutation of rice has often been used to disrupt EBE sequences, block the recognition and interaction between TALE proteins and their target, and reduce disease susceptibility [4,5]. In addition, plants actively defend themselves against pathogens through similar strategies.

The BLB resistance (*R*) gene *Xa21* from *Oryza longistaminata* L. was the first such resistance gene cloned from rice [6]. So far, researchers have found approximately 47 BLB *R* genes in cultivated and wild rice, among which 31 are dominant and 16 recessive [1,7,8]. Namely, *Xa1*, *Xa2/Xa31*, *Xa4*, *xa5*, *Xa7*, *Xa10*, *xa13*, *Xa14*, *Xa21*, *Xa23*, *Xa3/Xa26*, *Xa27*, *xa25*, *Xa27*, *xa41*, *Xa45*, and *xa44* were cloned [9], providing a rich genetic resource for breeding BLB-resistant rice. However, among the identified *R* genes, most have narrow antibacterial spectra. The few conferring broad-spectrum resistance, including *Xa4*, *Xa7*, *Xa21*, and *Xa23*, are widely used in cultivated rice breeding programs [9,10,11,12]. The recessive genes, such as *xa5*, *xa8*, and *xa13*, among others, exhibit broad antibacterial spectra but are difficult to apply to hybrid rice [13,14,15]. In addition to the co-evolution of *Xoo* and rice, many years of field cultivation have caused some disease-resistant rice varieties to lose resistance gradually [1]. Therefore, few genes are available for effective use in production, and exploring new resistance genes and identifying new varieties with broad-spectrum resistance for the control of BLB is critical. In addition to the above-mentioned major *R* genes, studies have also found many defense-response-related genes, including QTL, plant protection hormone synthesis-related, plant defense enzyme, and transcription factor family genes, which can regulate rice resistance to pathogens [16,17].

Among the major genes mentioned above, *Xa23*, which is derived from wild rice (*Oryza rufipogon*), has been extensively studied and widely used in rice-breeding programs [12,18,19,20,21]. *Xa23* is an executor *R* gene highly resistant to the physiological *Xoo* subspecies tested so far and is dominant and disease-resistant throughout the growth period. A particular TALE, *AvrXa23*, is widely present in tested physiological *Xoo* subspecies, and *AvrXa23* specifically bound the EBE in the *Xa23* promoter region, thereby activating *Xa23* expression and triggering a strong disease-resistance response. *AvrXa23* is present in all *Xoo* strains, including the highly pathogenic PXO99^A^ strain [12,22,23]. The *avrXa23* gene of wild-type PXO99^A^ was destroyed by using Tn5 transposition mutagenesis technology, and the resulting mutant, P99M2, overcame *Xa23* resistance [24]. Additionally, RNA-seq revealed the transcription factors and peroxidase genes associated with PXO99^A^ resistance. The wild-type *Xoo* strain PXO99^A^ and its mutant strain PH without any TALE were inoculated on susceptible rice JG30, and their differentially expressed genes (DEGs) were revealed by RNA-seq [25].

AH28 was screened from 185 native *Xoo* strains from different regions of China as highly virulent against CBB23 [1]. The AH28 strain does not contain the non-virulence *avrXa23* but contains the *tal7b* gene, which is similar to *avrXa23*. The *tal7b* gene does not have the non-virulence gene function of *avrXa23* and cannot stimulate the resistance of *Xa23*. The expression levels of resistance genes or resistance-related factors and metabolites in CBB23 infected with AH28 are unclear. In this study, RNA-seq and metabolomics techniques were used to analyze the DEGs of CBB23 infected by the highly pathogenic strain AH28 and hardly pathogenic strain PXO99^A^ and to screen genes related to BLB resistance in rice. At the same time, the metabolites were analyzed to clarify the BLB resistance mechanism in rice and enhance the understanding of the pathogenic mechanism of strain AH28 against CBB23.

## 2. Results

### 2.1. Resistance Reaction of Rice Inoculated with AH28 and PXO99^A^

**Figure 1:**
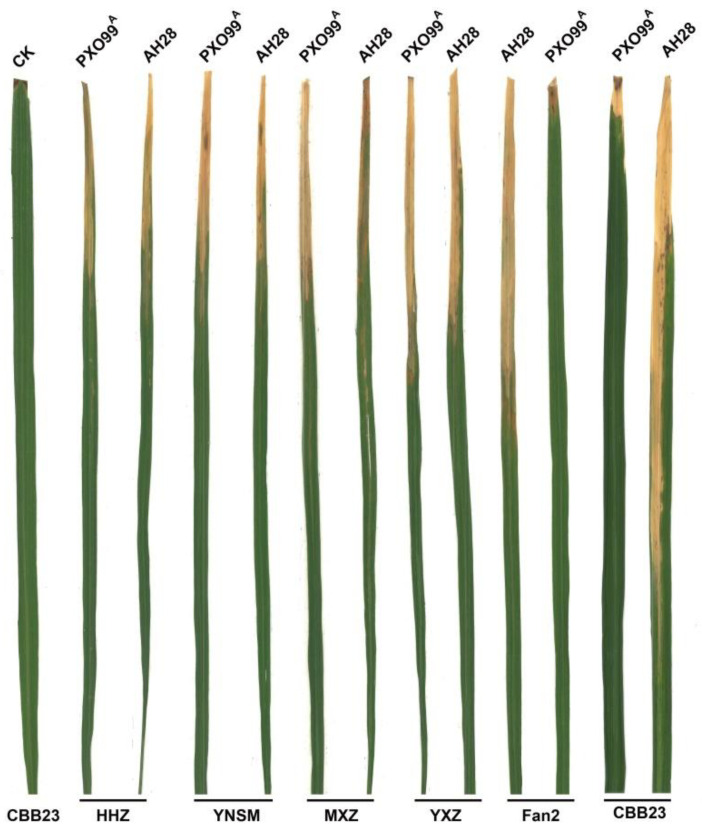
Comparison of *Xoo* pathogenicity of AH28 and PXO99^A^ in different rice varieties. CK was the control for uninfected bacteria. Both CBB23 and Fan2 contain *Xa23*, and the other varieties do not contain *Xa23*. HHZ, YNSM, MXZ, and YXZ are varieties that are widely cultivated in South China. Fan2 is a germplasm resource with high resistance to PXO99^A^ bred using KangbaiPR1 parent from the Guangdong Provincial Rice Genetic Resource Center.

The rice varieties CBB23, Huanghuazhan (HHZ), Yuenongsimiao (YNSM), Meixiangzhan (MXZ), Yuexiangzhan (YXZ), and Fan2 were inoculated with the *Xoo* strains AH28 and PXO99^A^ using the leaf-clipping method, and lesions were observed. The results showed that CBB23 and Fan2 were resistant to AH28 and PXO99^A^ (Figure 1). The lesion length of PXO99^A^ on CBB23 leaves was approximately 2.12 cm, while that of AH28 was approximately 25.65 cm (Table 1). The lesion length of PXO99^A^ on Fan2 leaves was approximately 2.08 cm, while that of AH28 was approximately 19.38 cm (Table 1). YNSM, MXZ, and YXZ were all susceptible to AH28 and PXO99^A^ after inoculation, while HHZ was moderately resistant. The lesion length of AH28 on CBB23 and Fan2 leaves was longer than that of PXO99^A^ after inoculation (Figure 1; Table 1), indicating that AH28 was more pathogenic to CBB23 and Fan2 than PXO99^A^. AH28 and PXO99^A^ had similar pathogenicity towards HHZ, YNSM, MXZ, and YXZ (Figure 1; Table 1).

### 2.2. DEG Analysis

**Figure 2:**
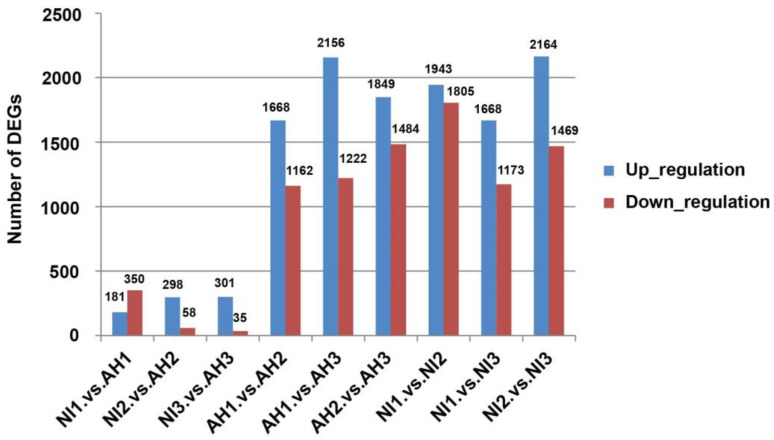
Statistical analysis of DEGs compared between different groups. The blue and red bars indicate up-regulated and down-regulated DEGs, respectively. Three biological replicates were made for each RNA-seq-treated sample.

In the early stage of inoculation, *Xoo* will secrete a large number of effectors into rice, interfere with the immune response of rice, achieve successful inoculation, and reproduce in rice leaves in the later stage, thus causing rice disease. At present, RNA-seq analysis of DEGs in rice after inoculation with *Xoo* mainly focuses on the late stage of infection, while there are few studies using RNA-seq analysis focusing on early response. In the preliminary experiment, *OsPR1a*, *OsPR1b*, *OsPR10b*, and other disease-resistance-related genes were significantly differentially expressed at 1 h after inoculation (hpi) and 1 d and 2 d after inoculation (dpi) with *Xoo*. Therefore, this study conducted RNA-seq analysis on the leaves of these three inoculation periods. After inoculating CBB23 with AH28 and PXO99^A^, infected leaves were selected at 1 hpi, 1 dpi, and 2 dpi for transcriptome sequencing and analysis. PXO99^A^-infected CBB23 leaf samples collected at 1 hpi, 1 dpi, and 2 dpi were named NI1, NI2, and NI3, respectively. AH28-infected CBB23 leaf samples collected at 1 hpi, 1 dpi, and 2 dpi were named AH1, AH2, and AH3, respectively. Three biological replicates were taken from each experimental group, and a total of 18 RNA-seq samples were analyzed. A total of 121.32 GB of clean data were obtained from RNA-seq analysis, with each sample contributing 5.70 GB and achieving a percentage of Q30 bases of 91.92% or above. The efficiency of comparison between clean reads of each sample and the reference genome ranged from 92.74 to 95.96%. The above results show that the sequencing quality and data volume were sufficient for the subsequent bioinformatics analysis. To further clarify which genes were involved in responding to *Xoo* after the inoculation of CBB23 with AH28 and PXO99^A^, the DEGs between samples infected with the two strains were analyzed after inoculation at each time point (Figure 2). A total of 7997 DEGs were detected (fold change ≥ 2 and false discovery rate < 0.01). In the three comparison groups of NI1 vs. AH1, NI2 vs. AH2, and NI3 vs. AH3, 531 (181 up-regulated and 350 down-regulated), 356 (298 up-regulated and 58 down-regulated), and 336 (301 up-regulated and 35 down-regulated) DEGs, respectively, were identified (Figure 2), for a total of 1010. There were more up-regulated than down-regulated genes. A large number of DEGs were detected in the comparison groups of AH1 vs. AH2, AH1 vs. AH3, AH2 vs. AH3, NI1 vs. NI2, NI1 vs. NI3, and NI2 vs. NI3, and there were more up-regulated than down-regulated genes in each group, especially in AH 1 vs. AH3 and NI2 vs. NI3.

### 2.3. DEGs in Different Pathways Analysis

To clarify the functional role of these DEGs in the AH28 and PXO99^A^ inoculation of CBB23, the gene ontology (GO) functional and Kyoto encyclopedia of genes and genomes (KEGG) pathway enrichment analyses were performed. Based on the principle of sequence similarity, each DEG matched at least one GO term and could be further refined into three domains: biological processes, cellular components, and molecular functions. In AH1 vs. AH2, AH1 vs. AH3, AH2 vs. AH3, NI2 vs. AH2, NI3 vs. AH3, NI1 vs. NI3, and NI2 vs. NI3, a large number of DEGs were enriched in a “cellular process” and “metabolic process” within biological processes. “Cellular anatomical entity” was the main enriched term within cellular components, and “catalytic activity” and “binding” were highly enriched within molecular functions. The number of up-regulated genes was significantly higher than that of down-regulated genes in all significantly enriched terms. In NI1 vs. AH1, the terms significantly enriched in the three ontologies were the same, but the number of up-regulated genes was significantly lower than that of down-regulated genes. In NI1 vs. NI2, the terms significantly enriched in the three ontologies were the same, but there was no significant difference between the numbers of up- and down-regulated genes.

**Figure 3:**
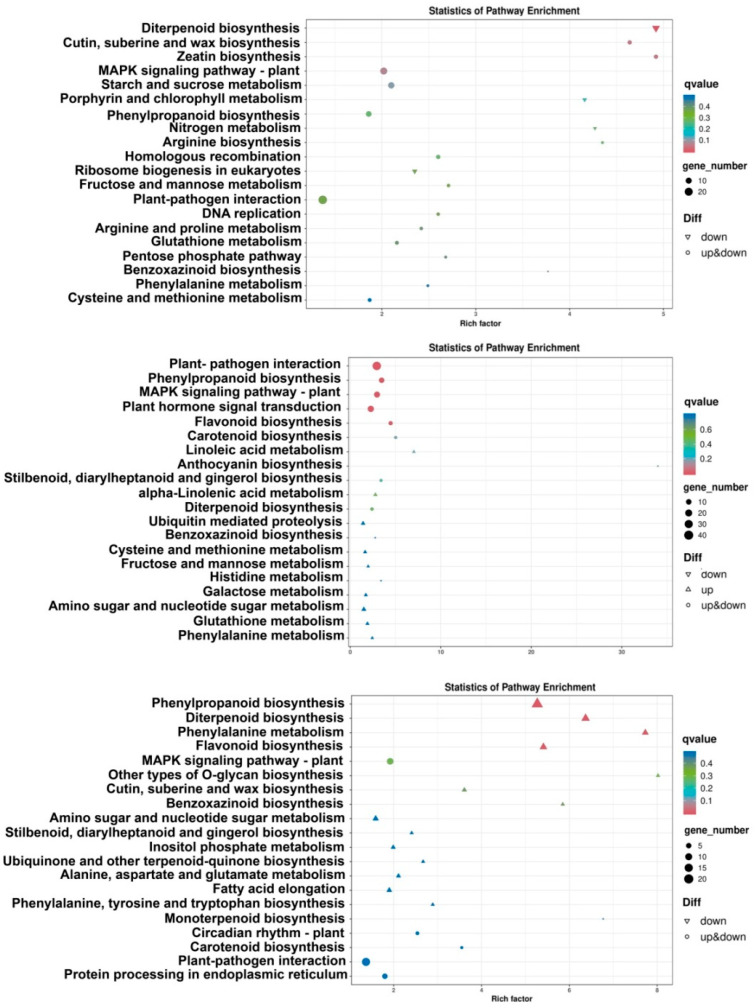
KEGG pathway enrichment bubble diagram of DEGs at NI1 vs. AH1 (**top**), NI2 vs. AH2 (**middle**), and NI3 vs. AH3 (**bottom**).

Most of the DEGs were subjected to KEGG pathway analysis (Figure 3). AH1 vs. AH2, AH2 vs. AH3, NI1 vs. AH1, NI1 vs. NI2, NI1 vs. NI3, NI2 vs. AH2, NI2 vs. NI3, and NI3 vs. AH3 were associated with plant hormone signal transduction, MAPK signaling pathway-plant, starch and sucrose metabolism, and plant–pathogen interaction pathway enrichment. In addition, in AH1 vs. AH3, there was a large amount of DEG enrichment in ribosome biogenesis in eukaryotes, carbon metabolism, and other pathways. In NI1 vs. NI2 and NI1 vs. NI3, protein processing in the endoplasmic reticulum, ribosome, carbon metabolism, and phenylpropanoid biosynthesis pathway were enriched. Phenylpropanoid biosynthesis was also highly enriched in NI2 vs. AH2, NI2 vs. NI3, and NI3 vs. AH3. These results indicated that a large number of DEGs were significantly enriched in several functional classes and metabolic pathways (plant hormone signal transduction, MAPK signaling pathway-plant, starch and sucrose metabolism, plant–pathogen interaction, carbon metabolism, and phenylpropancid biosynthesis) in CBB23 in response to inoculation by two *Xoo* strains.

### 2.4. DEGs Are Closely Related to Disease Resistance

**Figure 4:**
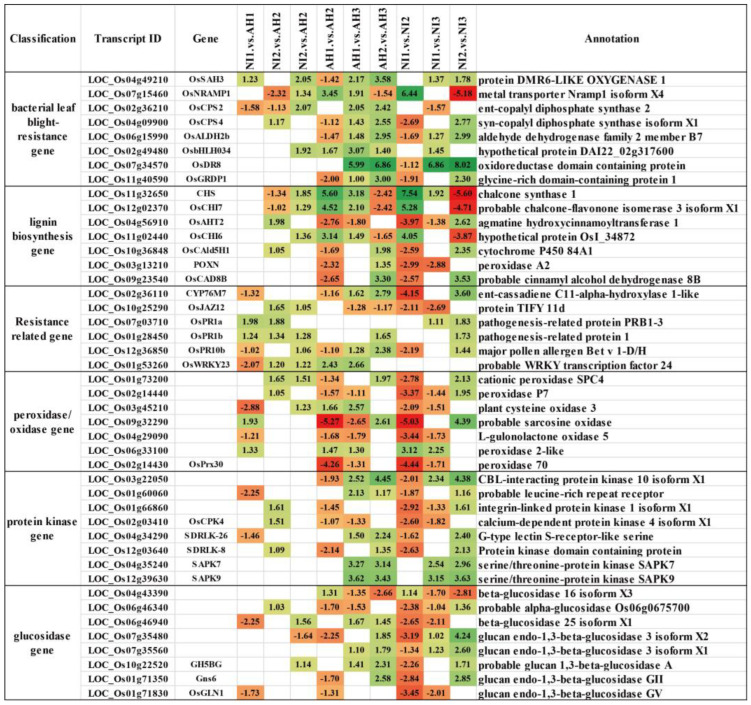
The partial differential expression of the comparison between different groups. The number is the log_2_FoldChange ratio between the comparison groups, with red representing down-regulation, green representing up-regulation, and white representing no significant difference.

Database sequence and functional comparisons with the annotated genes revealed that among all DEGs detected in this experiment, a large number related to disease resistance and course were significantly differentially expressed in samples at different time points, including cloned rice BLB resistance, peroxidase, lignin synthesis, NBS-LRR, pathology-related, protein kinase, glucosidase, and other coding genes. Eighty-two cloned resistance genes were differentially expressed, among which 40, 47, and 10 were directly related to BLB, rice blast, and sheath blight, respectively (Figure 4). *OsSAH3*, *OsNRAMP1*, *OsS5H2*, *OsALDH2b*, *OsPAL2*, and *OsPAL4* were related to BLB and rice blast, while *Xa23* was not differentially expressed. *OsPR10b*, *CYP76M7*, *OsJAZ12*, *OsPR1b*, *OsWRKY23*, *OsWRKY4*, *OsSERK3*, and *OsPR10* were associated with disease resistance. Lignin can regulate the plant immune system and improve disease resistance in rice. In this study, 18 genes related to lignin synthesis were detected, including *OsCHS1*, *OsCHI7*, *OsCHI6*, *OsCAld5H1*, *OsGF14b*, and *NAC028*, among others. Five and nine differentially expressed NBS-LRR and SWEET genes, respectively, were detected, among which *DEPG1*, *OsSWEET15,* and *Xa25* had been cloned. Phenylalanine aminolyase (PAL), peroxidase (PO), chitinase (CHT), and glucosidase are involved in plant disease resistance. In this study, 7, 42, 12, and 41 DEGs, respectively, encoding related proteases were detected. *OsPrx30*, *OsCEBiP*, *OsCHI11*, *OsAPx8*, *OsCEBiP*, and *OsCEBiP* were cloned genes related to disease resistance. Protein kinases play an important role in plant resistance to disease. In this study, 278 protein kinases were differentially expressed, among which *OsCPK4* and *OsFLS2*, which are related to leaf stem disease resistance, had been cloned. Plant hormones, such as salicylic and jasmonic acids, are involved in the interactions between plants and pathogens. In this study, seven and thirteen genes, respectively, related to hormone synthesis and metabolism were detected, including *OsSGT1*, *OsPAL3*, *OsPAL4,* and *ONAC131*, which are related to salicylic acid synthesis and metabolism, and *OsAOS3*, *OsJAZ13*, *ONAC131*, *OsPAL4*, *OsNAP*, and *OsWRKY85*, which are related to jasmonic acid synthesis and metabolism. In addition to the above DEGs related to disease resistance, genes encoding glutathione peroxidase and similar genes, metallothionein genes, laccase protein precursors, alcohol oxidase, and oxidoreductase genes were also differentially expressed.

### 2.5. Transcription Factor (TF) Analysis

**Figure 5:**
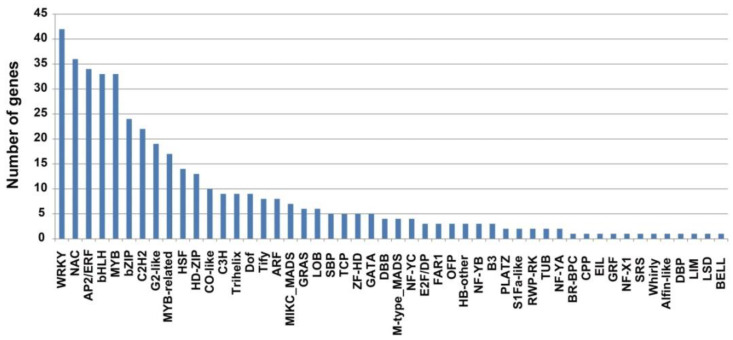
The number of differentially expressed transcription factors of CBB23 in response to the two *Xoo* strains.

**Figure 6:**
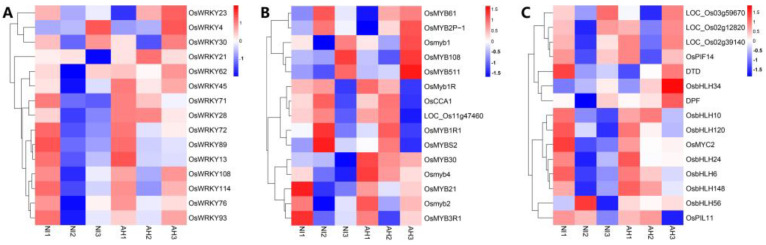
Heatmaps of differentially expressed (**A**) WRKY, (**B**) MYB, and (**C**) bHLH transcription factors by CBB23 in response to the two *Xoo* strains. After the value is homogenized, the color depth indicates the difference between the gene expression and the mean value. The red column represents up-regulation, and the blue column represents down-regulation.

PlantTFDB (the Plant Transcription Factor Database) was used to predict transcription factors (TFs) associated with DEGs and annotate the DEGs to families. The analysis showed that the DEGs in this study were annotated to 50 TF families, with a total of 431 TFs (Figure 5). The WRKY family has the largest number of members involved in plant disease resistance, with 42, followed by the NAC and bHLH families, with 36 and 33, respectively. The MYB, bZIP, WRKY, AP2/ERF, and NAC TFs were all expressed to varying degrees, and six transcription factors showed greater frequencies of associated DEGs in the nine comparison groups. They belonged to the WRKY, NAC, MYB, bHLH, NAC, and AP2/ERF families (Figure 5 and Figure 6). Among all the differentially expressed TFs noted, 18 cloned TFs were directly related to BLB, including the WRKY TFs *OsWRKY71*, *OsWRKY30*, *OsWRKY67*, *OsWRKY53*, *OsWRKY76*, *OsWRKY62*, *OsWRKY81*, *OsWRKY13*, *OsWRKY45*, and *OsWRKY72*, among others; the MYB TFs *OsMYB5P*, *Osmyb4*, and *OsMYB21*, among others; and the bHLH transcription factors *OsbHLH034*, *OsbHLH6*, and *OsMYC2*, among others (Figure 5 and Figure 6). There were differences in *OsWRKY71* expression in the NI1 vs. NI2, NI1 vs. NI3, AH1 vs. AH3, and NI2 vs. AH2 comparisons, among which *OSWRKY71* expression was only up-regulated in NI2 vs. AH2. There were differences in *OsWRKY30* expression in NI1 vs. NI2, NI2 vs. NI3, AH1 vs. AH2, and AH2 vs. AH3, among which *OSWRKY30* expression was up-regulated in NI2 vs. NI3 and AH2 vs. AH3. These data also indicated that a large number of TFs were involved in the response after the *Xoo* infection, among which WRKY, bHLH, and MYB family TFs played an important role in the resistance of rice CBB23 against the *Xoo* strains.

### 2.6. qPCR Verification and Analysis of DEGs in the Transcriptome

**Figure 7:**
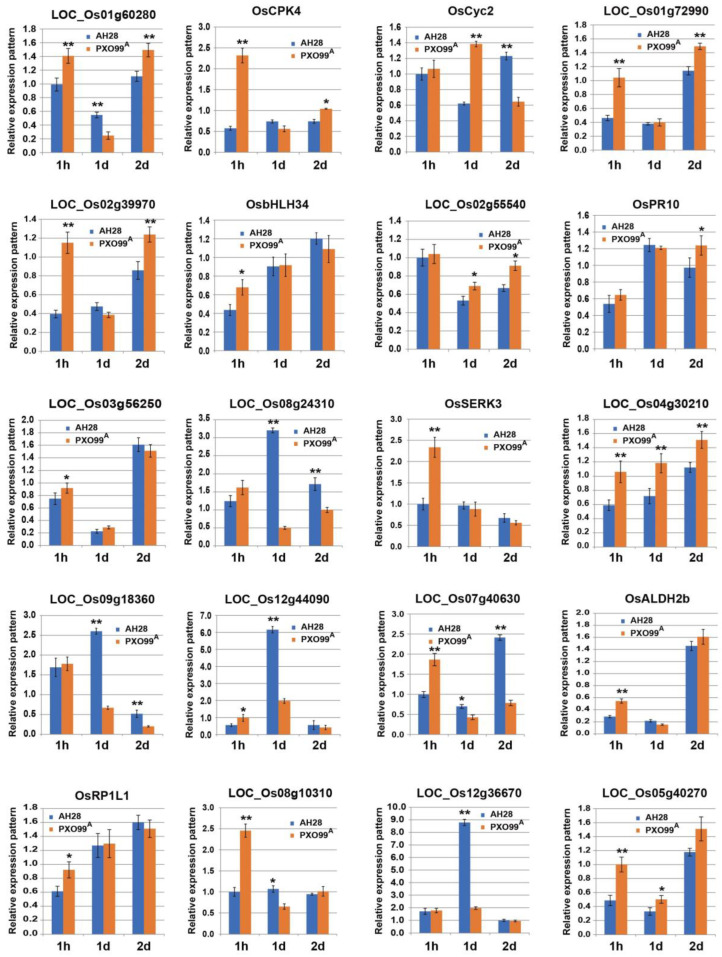
qRT-PCR-based validation of DEGs in response to AH28 and PXO99^A^ at different time intervals. The DEGs were randomly selected for qRT-PCR. Values are mean ± SD from three biological replicates. 1 h, 1 d, and 2 d refer to 1 h after inoculation (hpi), 1d after inoculation (dpi) and 2d after inoculation (dpi), respectively. * and ** present significant differences at *p* < 0.05 and *p* < 0.01 between the expression of leaves infected by AH28 and PXO99^A^, respectively.

To verify the reliability of the RNA-seq results, 20 DEGs were randomly selected for transcriptome sequencing in qRT-PCR experiments, including *OsCPK4*, *OsCyc2*, *OsbHLH34*, *OsPR10*, *OsSERK3*, *OsALDH2b*, and *OsRP1L1*, and other DEGs were significantly differentially expressed in the plant–pathogen interaction pathway. Gene-specific primers were designed for qRT-PCR amplification, and the results are shown in Figure 7. The relative expression levels of genes were consistent with the results of RNA-seq, which indicated that the RNA-seq data obtained in this study were reliable.

### 2.7. Metabolome Analysis of CBB23 in Response to Disease Resistance

**Figure 8:**
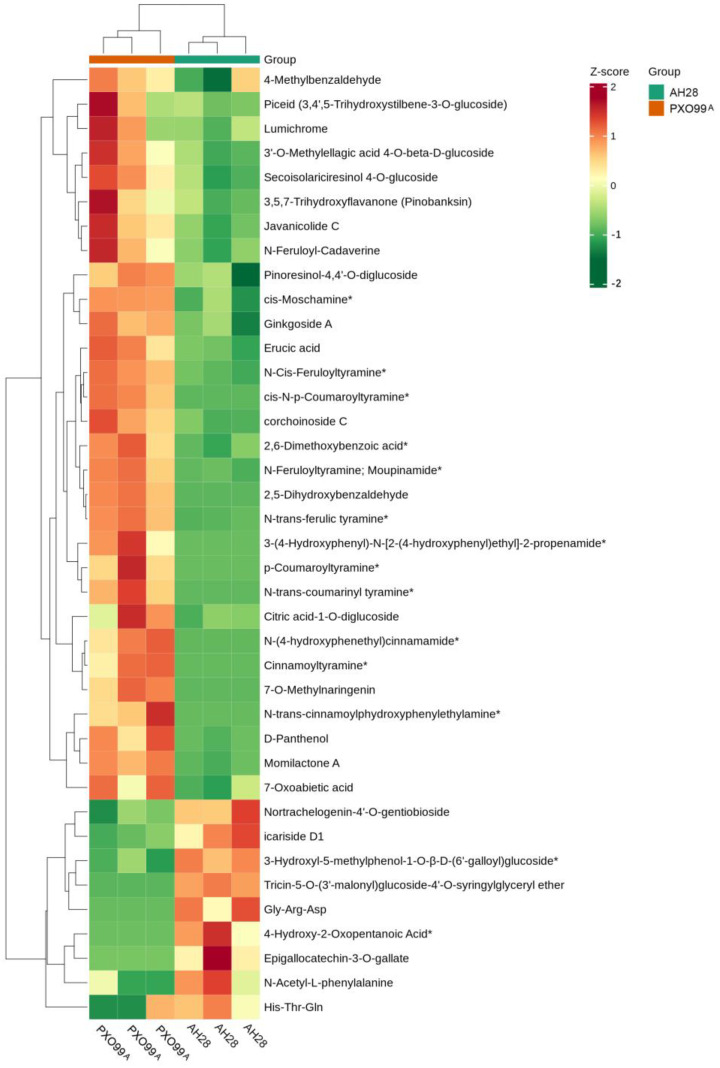
A heat map of the relative differences in multiple differential metabolites detected using GC–MS between the two bacterial strains infected with CBB23 at 2 dpi. The red column represents up-regulation, and the green column represents down-regulation.

In order to understand the changes in metabolites in response to disease resistance after inoculation in CBB23 with different strains of BLB, metabolome analysis was performed on the leaves of the two bacterial strains infected with CBB23 at 2 dpi (Figure 8). Through analysis, it was found that the top three types of differential metabolites were alkaloids, flavonoids, and amino acids and their derivatives, and the three types of differential metabolites accounted for about 51.28% of the total differential metabolites, among which the alkaloid differential metabolites accounted for about 33.33% of the total differential metabolites. The contents of all the different alkaloids and terpenoids detected in AH28-infected leaves were significantly lower than those in PXO99^A^-infected leaves, such as Cinnamoyltyramine, N-Cis-Feruloyltyramine, cis-N-p-Coumaroyltyramine, 7-Oxoabietic acid and Javanicolide C, etc. (Figure 8). On the contrary, the contents of all amino acids and their derivatives in AH28-infected leaves were significantly higher than those in the leaves infected by PXO99^A^, such as N-Acetyl-L-phenylalanine, Gly-Arg-Asp, and His-Thr-Gln. The contents of flavonoids, lignin, coumarins, phenolic acids, lipids, and other metabolites were different in the two infected leaves. In this study, the differential metabolite content detected in AH28-infected leaves was mostly lower than that of leaves infected with PXO99^A^. The KEGG pathway analysis of the detected differential metabolites showed that the highest proportion of metabolic pathways was 36.36%, followed by the phenylalanine metabolic pathway, flavonoid biosynthesis pathway, and secondary metabolite biosynthesis pathway.

## 3. Discussion

### 3.1. The Exploration of New Genes Plays an Important Role in the Prevention and Control of Virulent Bacterial Strains

Rice BLB is a bacterial and perennial epidemic disease in southern China. At present, many factors are related to BLB resistance, and resistance-related TFs of different families interact with each other, such that rice has different specific or broad-spectrum resistances. For example, some WRKY genes can regulate the PR genes of rice and then activate plant hormones or other disease-resistance factors [17]. In addition, disease-resistance factors are also affected by major disease-resistance genes that jointly resist inoculation by *Xoo*. For example, *Xa21* can bind to the WRKY TF *OsWRKY62* to negatively regulate rice disease resistance [26]. In addition, *Xa21* can also interact with auxin-like protein XB21 to improve resistance to *Xoo* [27]. Therefore, the interaction between the same rice resistance gene and different resistance factors can produce completely different immune responses. In this study, CBB23 containing *Xa23* had high resistance to bacteria with PXO99^A^. In contrast, MXZ, YNSM, and YXZ, which did not contain *Xa23*, were highly susceptible to both *Xoo* strains; however, after inoculation with AH28, the lesion size of MXZ, YNSM, and YXZ was significantly smaller than that of CBB23 (Figure 1, Table 1). This result indicated that MXZ, YNSM, YXZ, and other strains may have other resistance genes or resistance-related factors different from those of CBB23 that can be induced by AH28 to reduce the toxicity of AH28. At the same time, HHZ without *Xa23* showed middle sensitivity to both strains, which further indicated that resistance genes or resistance-related factors cooperated with each other to make rice have different specific or broad-spectrum resistance. A polymerized line containing both the *xa5* and *Xa4* was developed by using the molecular marker-assisted selection (MAS) of polygene polymerization [28]. Four resistance genes, *Xa7*, *Xa21*, *Xa22*, and *Xa23*, were polymerized into Huahui 1035, and the progeny showed different levels of resistance to 11 representative bacterial isolates from China [20]. The simultaneous introduction of *Xa4*, *Xa21*, and *Xa27* into the rice restorer lines of Mianhui 725 or 9311 at the same time significantly improved resistance to BLB [11]. A *japonica* rice variety, “Tainung82”, containing five genes, *Xa4*, *xa5*, *Xa7*, *xa13*, and *Xa21*, was successfully bred using MAS, which not only greatly improved the resistance of the variety to BLB but also maintained its original high yield and excellent quality [29]. *Xa38* was introduced into the rice maintainer line APMS 6B, which improved resistance while retaining good agronomic traits [30]. In this study, a number of rice varieties widely cultivated in South China were infected with AH28 and showed high susceptibility. Taken together, the results of this study suggest that the aggregation of multiple resistance genes may reduce the toxicity of AH28. However, polygene aggregation does not necessarily enhance disease resistance. It found that *xa5* attenuates *Xa27*-mediated *Xoo* resistance [31]. The induced expression of *Xa23* by PXO99^A^ is lost after *xa5* homozygous mutation [32]. Therefore, the key to enhancing the resistance of rice to BLB is to explore the genes resistant to AH28 and rationally pyramiding and utilize the resistance genes in different strains.

### 3.2. Possible Genes Involved in the Resistance Response of Plants Containing xa23 to Xoo Strains

The *Xa23* promoter region contains the EBE of *AvrXa23*, which can be specifically bound by *AvrXa23* in *Xoo* and activate *Xa23* transcription to make the plant resistant to disease [18,33]. Although the susceptible allele *xa23* has the same coding sequence as *Xa23*, its promoter region lacks the EBE of *AvrXa23* elements and cannot be activated and expressed by *AvrXa23*, showing a susceptible phenotype [12]. The AH28 used in this study can make *Xa23* susceptible. Transcriptome analysis was used to identify the resistance genes or resistance-related factors involved in the immune response of rice infected by AH28 and PXO99^A^ after they infected CBB23. In order to reveal the molecular mechanism of DEGs in rice resistance to BLB, it was found that most of the genes were up-regulated, indicating that the expression of rice disease-resistance-related genes was activated after inoculation by the two strains. In this study, metabolome analysis showed that the contents of a large number of different metabolites, including alkaloids and terpenoids, were significantly lower in leaves infected by AH28 than in leaves infected by PXO99^A^ (Figure 8).

A large number of cloned genes related to BLB resistance in rice were differentially expressed among different groups, but among the 17 cloned BLB resistance genes mentioned above, only *Xa25* was differentially expressed in this study. *Xa25* was differentially expressed in the NI1 vs. NI2, NI2 vs. NI3, and AH1 vs. AH2 groups. In this study, the differential expression of five NBS-LRR genes, nine SWEET gene families, and seven phenylalanine ammoniase genes were detected, including in cloned *OsRP1L1*, *OsPAL6*, *OsPAL1*, *OsPAL3*, *OsPAL4*, *OsSWEET4*, *OSRP1L1*, *OSPAL6*, *OspAL1*, *OSSWEET4*, *OsSWEET15*, *Xa25*, *OsSWEET14,* and *OsSWEET2a* [34,35,36,37,38,39]. The differential expression of 278 genes that encode protein kinase was detected, in addition to 41 genes encoding glucosidase, 93 genes encoding peroxidase, and 14 lignin-related genes (Figure 4). The types of proteins encoded by these detected DEGs are reportedly involved in resistance to rice BLB. KEGG analysis of differential metabolites in metabolome showed that a large number of differential metabolites were metabolic pathways, phenylalanine metabolism, and flavonoid biosynthesis pathways. The types of proteins encoded by these detected DEGs and the differential metabolites have been reported to be involved in the resistance of rice BLB and other diseases [40,41].

In plants, the SWEET protein, as a sugar transport protein, plays an important role in plant reproductive development, plant–pathogen interaction, aging, and stress. In the process of infecting rice plants, TALE proteins bind to EBE of the SWEET gene promoter, which activates SWEET gene expression, thus making the rice susceptible to disease. Rice contains more than 20 SWEET genes, some of which govern BLB susceptibility [35,42,43]. Three BLB resistance genes in rice belong to the SWEET gene family: *xa13* [44], *xa25* [45], and *xa41 (t)* [46]. In this study, the differential expression of cloned genes, such as *OsSWEET4*, *OsSWEET15*, *Xa25*, *OsSWEET14*, and *OsSWEET2a*, was detected (Figure 5 and Figure 6). In addition, *OsSWEET1a*, *OsSWEET2b*, *OsSWEET3b*, and *OsSWEET16* were also detected. *OsSWEET2b* and *OsSWEET3b* were differentially expressed in the NI1 vs. NI2, NI2 vs. NI3, AH1 vs. AH2, and AH2 vs. AH3 groups. Their function and the molecular mechanisms in the immune response of rice to BLB need to be further verified.

Transcription factors play an important role in plant disease resistance. The NAC TF family gene *OsNAC60* and the MYB TF family gene *OsMYB30* regulate the disease resistance of rice to several *M. oryzae* subspecies [47,48]. The WRKY TF family genes *OsWRKY45* and *OsWRKY67* are involved in regulating rice resistance to blast and blight [49,50]. *OsWRKY3* and *OsWRKY71* acted on the expression of resistance genes, such as *OsNPR1* and *OsPR1b*, and had significant inhibitory effects on BLB [51]. *OsWRKY13* can directly or indirectly regulate the expression of the upper and lower functional genes of salicylic and jasmonic acid metabolism and enhance resistance to BLB and blast in rice during the whole growth period [52]. *WRKY62* negatively regulates the resistance mediated by *Xa21*, a major gene for resistance to BLB, and inhibits the activation of defense-related genes in rice after inoculation [53]. In this study, differences in the expression of 42 WRKY family transcription factors were detected between different groups (Figure 5 and Figure 6). The cloned genes included *OsWRKY71*, *OsWRKY30*, *OsWRKY67*, *OsWRKY53*, *OsWRKY76*, *OsWRKY62*, *OsWRKY81*, *OsWRKY13*, *OsWRKY45*, *OsWRKY72*, and so on. The transcriptional regulatory network in which many TFs participate is large and complex. Therefore, the analysis of disease resistance signaling pathways regulated by different TFs will provide a theoretical basis for molecular breeding for disease resistance.

Rice can sense the changes in the cell wall after infection by *Xoo* and rapidly transmit the signals into the cell through various ways, triggering the reconstruction of transcription level and thus activating the disease resistance signal pathway. At the same time, it synthesizes a large number of secondary metabolites to kill pathogenic microorganisms and resist the invasion of *Xoo*. The metabolomics analysis of the changes in metabolites after the invasion of pathogens can provide a new understanding of the mechanism of plant disease infection. Metabolomics showed that resistant varieties increased secondary metabolite production through the shikimic acid pathway and promoted resistance to brown planthopper [54]. Metabolomics and transcriptomics were used to study the differences in resistance of different varieties to *Xoo*. The results showed that the biosynthesis of acetophenone, lutein, fatty acids, alkaloids, glutathione, carbohydrates, and lipids of *Xoo*-invaded rice leaves was an important reason for the differences in resistance of rice varieties [55]. Phytoalexin (PA), also known as plant defensins or plant antitoxins, is a class of secondary metabolites that play a defensive role when plants are infected by pathogens and are synthesized and accumulated by their own metabolic pathways [56]. PA includes a variety of compounds, including flavonoids, terpenoids, alkaloids, and glycosteroids, which are widely reported to be involved in plant disease-resistant immune responses. It has been reported that the MAPK signaling pathway, amino acid metabolism, TCA cycle, and other metabolic pathways are closely related to the vegetative growth and development and pathogenicity of plant pathogens [57,58]. Amino acid metabolism plays an important role in the infection cycle and pathogenicity of pathogens [57,58]. Many secondary metabolites, such as alkaloids, phenols, and flavonoids, play an important role in plant defense response [59]. In this study, metabolome analysis found that alkaloids, flavonoids, amino acids, and their derivatives were the main differential metabolites, and more differential metabolites were found in metabolic pathways, phenylalanine metabolic pathways, flavonoid biosynthesis pathways, and secondary metabolites biosynthesis pathways. Meanwhile, differences were also detected in metabolites such as lignin, coumarin, phenolic acids, and lipids. In the study, metabolites such as alkaloids and amino acid metabolism were involved in the resistance reaction of rice against bacterial infection. In rice varieties containing *Xa23*, alkaloids, flavonoids, amino acids, and their derivatives metabolites were mainly involved in the resistance reaction. A large number of genes are involved in the regulation of plants in the process of coping with stress, including changes in plant morphology, synthesis or degradation of related metabolites, and increase or decrease of hormone levels [56,57,58,59]. In this study, transcriptome analysis showed that a large number of DEGs were significantly enriched in plant hormone signal transduction, MAPK signaling pathways, starch and sucrose metabolism, plant–pathogen interactions, carbon metabolism, and phenylpropionic acid biosynthesis. DEGs related to disease course, plant protection hormone synthesis, phenylalanine aminlyase, genes with typical resistance domains such as NBS-LRR, and transcription factors were detected. DEGs are highly enriched in related pathways, such as plant–pathogen interaction, plant hormone signaling, and phenylpropionic acid biosynthesis, which is consistent with the enrichment of differential metabolites in phenylalanine metabolism, flavonoid biosynthesis, and secondary metabolite biosynthesis.

## 4. Materials and Methods

### 4.1. Rice and Bacterial Strains

CBB23 contains *Xa23*, a gene related to BLB resistance. HHZ, YNSM, MXZ, and YXZ are cultivated varieties widely cultivated in South China. Fan2 is a germplasm resource with high resistance to PXO99^A^ bred using KangbaiPR1 parent from the Guangdong Provincial Rice Genetic Resource Center. All plants were grown in light incubators under conventional water and fertilizer management. The AH28 strain is a wild-type bacterial strain that can overcome *Xa23*-mediated resistance and was provided by Gongyou Chen’s laboratory at Shanghai Jiao Tong University (Shanghai, China) [1]. PXO99^A^ is a model strain for studying *Xa23* and was inoculated into rice containing the *Xa23* resistance gene. All strains were stored in glycerol at −80 °C. The two *Xoo* strains were cultivated on nutrient broth medium at 28 °C for 2 d in the dark. Inoculated bacteria solutions were prepared with sterile water, and their concentration was adjusted to OD_600_ = 1.0.

### 4.2. Inoculation and Investigation of BLB

AH28 and PXO99^A^ inoculation and identification were performed on CBB23, HHZ, YNSM, MXZ, YXZ, and Fan2, and the plants were inoculated during the 6th week of the seedling stage using the artificial leaf-cutting inoculation method [60]. Rice seedlings with healthy growth states, consistent growth, normal leaf colors, and no spots were selected as experimental inoculation objects, and each plant was inoculated in 2 unfolding leaves. Inoculated bacteria were prepared with sterilized water and inoculated with bacteria solution with OD_600_ (600 nm optical density) of 1.0. Lesion length was measured at 14–20 dpi, when the disease of the infected varieties tended to be stable. A lesion length of <3 cm indicated disease resistance (R), 3–5 cm indicated medium resistance (MR), 6–9 cm indicated medium sensitivity (MS), and >10 cm indicated disease sensitivity (S) [61].

### 4.3. Sample Preparation, Sequencing, and Analysis of RNA-seq

AH28 and PXO99^A^ were inoculated into CBB23 using the above inoculation methods. Samples were taken at 1 hpi, 1 dpi, and 2 dpi. Inoculated leaves from 5 plants were collected at each time point, mixed into one sample, and put into centrifuge tubes. Three biological replicates were taken from each sample. The removed samples were immediately placed into liquid nitrogen for quick freezing and stored at −80 °C for RNA extraction and transcriptome sequencing. The total RNA was separated using TRIzol (Invitrogen, Carlsbad, CA, USA) according to the manufacturer’s protocol. The quality and quantity of extracted RNA were measured using 1% agarose gel and Agilent 2100 Bioanalyzer (Agilent Technologies, Santa Clara, CA, USA). The obtained high-quality RNA was sent to the company (Biomarker Technologies Co., Ltd., Qingdao, China) for library construction. After RNA extraction and purification and library construction, next-generation sequencing was based on the Illumina sequencing platform.

### 4.4. Analysis of DEGs and Their Functional Enrichment

DESeq was used to analyze differences in gene expression between the two groups of samples. The conditions for screening DEGs were as follows: expression difference multiple |log_2_Fold Change| ≥ 1, with significant *p*-value ≤ 0.05. The topGO was used for GO enrichment analysis. During the analysis, the DEGs annotated by GO terms were used to construct a gene list and gene number for each term, and *p*-values were calculated by the hypergeometric distribution., so as to determine the main biological functions performed by DEGs. KEGG enrichment analysis of DEGs was performed using the KEGG orthology-based annotation system (KOBAS) to reveal molecular interaction networks and metabolic pathways. The KEGG calculation method was the same as that for GO enrichment analysis.

### 4.5. RNA Extraction and qRT-PCR Detection

Total RNA was extracted from rice samples using TRIzol (Invitrogen, Carlsbad, CA, USA). Total RNA (500 ng) was obtained for reverse transcription, and RT-PCR was performed using the PrimerScript™ RT Reagent Kit (Takara Bio Inc., Kusatsu, Japan). qRT-PCR was performed using the SYBR Premix Ex TaqTM II kit (Takara) according to the manufacturer’s instructions, with the EF1α gene as the internal control. Three techniques were repeated for each reaction operation, and the total reaction mixture was 10 μL. The reaction conditions were as follows: 95 °C for 30 s, 95 °C for 10 s, 60 °C for 30 s, and 72 °C for 20 s for a total of 40 cycles. The relative expression level of genes was calculated using the 2^−ΔΔCT^ method [62]. The primers used for qPCR are listed in Supplemental Table S1.

### 4.6. Metabolome Sample Preparation and Metabolomics Analysis

AH28 and PXO99^A^ were inoculated to CBB23 using the above inoculation methods. Samples were taken at 2 dpi and then freeze-dried using a vacuum freeze-dryer (Scientz-100F, Scientz, Ningbo, China). The freeze-dried samples were crushed using a mixing mill (MM 400, Retsch, Haan, Germany) with zirconia beads for 1.5 min at 30 Hz. An aliquot of 100 mg of powder was then extracted overnight at 4 °C with 1.0 mL of 70% aqueous methanol containing 0.1 mg L^−1^ lidocaine (internal standard) before analysis using an LC-ESI-MS/MS system [63]. The chromatographic conditions were DB-5MS capillary column (30 m × 0.25 mm × 0.25 μm, Agilent J&W Scientific, Folsom, CA, USA), high purity helium carrier gas, flow rate 1.0 mL × min^−1^, and inlet temperature 260 °C. The sample size was 1 μL, the shunt ratio was 4:1, and the solvent delay was 5 min. The mass spectrum conditions were electron bombardment ion source (EI), ion source temperature 230 °C, quaternary bar temperature 150 °C, and electron energy 70 eV. The scanning mode was full scanning mode, and the quality scanning range was 50–500 m/z. One sample was inserted into every 9 analysis sample to examine the repeatability of the entire analysis process. GC/MS was used to detect the raw data by converting Analysis Base File Converter software (version 1.1.0.0) analysis files, importing MS-DIAL preprocessing software (version 4.9), and removing background noise. The characterization of metabolites was achieved by matching the similarity of retention time, precise molecular mass, mass charge ratio and mass spectral response intensity (peak area) with the database. All metabolite data were log_2_-transformed for statistical analysis to improve normality. The p- and fold-change values were set to 0.05 and 2.0, respectively, to screen for differentially accumulated metabolites.

## 5. Conclusions

In this study, differences in DEG and metabolite contents were analyzed after the inoculation of CBB23 by AH28 and PXO99^A^. A total of 7997 DEGs were detected, among which more genes were up- than down-regulated. The DEGs contained a large number of cloned genes related to BLB resistance, as well as uncloned genes related to BLB resistance. The main genes were peroxidase, protein kinase, glucosidase, and other coding genes, as well as genes related to lignin synthesis, salicylic acid, jasmonic acid, and secondary metabolites. At the same time, genes associated with BLB, including NBS-LRR, SWEET, and phenylalanine ammoniase genes, were also detected. A large number of TFs were also observed to participate in the BLB resistance, mainly WRKY, NAC, AP2/ERF, bHLH, and MYB family factors. Additionally, metabolomics analysis showed that a large number of alkaloids and terpenoids significantly decreased, while the content of amino acids and their derivatives significantly increased after AH28 inoculation of CBB23. This study provides a theoretical basis for gene cloning and molecular mechanism research related to BLB resistance and provides new disease resistance resources for rice breeding.

## Figures and Tables

**Table 1:** The results of lesion lengths of AH28 and PXO99^A^ inoculated with different rice materials.

Variety	PXO99^A^			AH28		
	Lesion Length (cm)	SD (cm)	Phenotypes	Lesion Length (cm)	SD (cm)	Phenotypes
CBB23	2.12	0.67	R	25.65	5.87	S
HHZ	9.50	3.39	MS	9.33	3.42	MS
YNSM	10.73	1.54	S	10.41	2.22	S
MXZ	15.53	2.16	S	17.98	3.67	S
YXZ	14.78	2.28	S	13.70	3.67	S
Fan2	2.08	0.53	R	19.38	3.94	S

Lesion length < 3 cm indicated disease resistance (R), 6–9 cm indicated medium sensitivity (MS), and >10 cm indicated disease sensitivity (S). Values are the mean from 10 biological replicates.

## Data Availability

The accession number of the original transcriptomic data on NCBI Sequence Read Archive is PRJNA1103348, and the identifier of the original metabolomic data on the EMBL-EBI MetaboLights database is MTBLS10047.

## Supplementary Materials

The following supporting information can be downloaded at: https://www.mdpi.com/article/10.3390/plants13101411/s1, Table S1 Primer pairs used for qPCR.
